# Prohibitin Attenuates Oxidative Stress and Extracellular Matrix Accumulation in Renal Interstitial Fibrosis Disease

**DOI:** 10.1371/journal.pone.0077187

**Published:** 2013-10-25

**Authors:** Tian-Biao Zhou, Yuan-Han Qin, Feng-Ying Lei, Wei-Fang Huang, Gregor P. C. Drummen

**Affiliations:** 1 Department of Pediatric Nephrology, the First Affiliated Hospital of GuangXi Medical University, NanNing, China; 2 Department of Nephrology, the Sixth Affiliated Hospital of Sun Yat-Sen University, Guangzhou, China; 3 Cellular Stress and Ageing Program, Bionanoscience and Bio-Imaging Program, Bio&Nano-Solutions, Düsseldorf, Germany; Institut National de la Santé et de la Recherche Médicale, France

## Abstract

Prohibitin is an evolutionary conserved and pleiotropic protein that has been implicated in various cellular functions, including proliferation, tumour suppression, apoptosis, transcription, and mitochondrial protein folding. Both prohibitin over- and under-expression have been implicated in various diseases and cell types. We recently demonstrated that prohibitin down-regulation results in increased renal interstitial fibrosis (RIF). Here we investigated the role of oxidative stress and prohibitin expression in RIF in unilateral ureteral obstructed rats. Lentivirus-based delivery vectors were used to knockdown or over-express prohibitin. Our results show that increased prohibitin expression was negatively correlated with the RIF index, reactive oxygen species, malon dialdehyde, transforming growth factor β1, collagen IV, fibronectin, and cell apoptosis index. In conclusion, we postulate that prohibitin acts as a positive regulator of mechanisms that counteract oxidative stress and extracellular matrix accumulation and therefore has an antioxidative effect.

## Introduction

Renal interstitial fibrosis (RIF) is characterized by the extensive and progressive accumulation of extracellular matrix (ECM) [1], [2]. RIF is a common denominator in virtually all forms of chronic kidney disease [3], regardless of the aetiology of the primary renal syndrome [4]. Additionally, interstitial fibrosis is the primary morphologic predictor of clinical outcome and is tightly linked to disease progression [4], [5].

Prohibitin (PHB) is ubiquitously expressed in a variety of cell types and multiple cellular compartments, including the mitochondria, nucleus, and the plasma membrane [6]. PHB is an evolutionary highly conserved protein [7] that has been implicated in cell cycle control, tumour suppression, and in the controlled inhibition of DNA synthesis initiation [8]. Furthermore, several lines of emerging evidence suggest that PHB expression or the lack thereof might be a factor in proliferation, differentiation, senescence, ageing, oxidative stress, necrosis and apoptosis. For instance, the PHB content is inversely associated with cell proliferation, but distinctly increases during granulosa cell differentiation as well as in earlier events of apoptosis in a temperature-sensitive granulosa cell line [9]. Gene interference with PHB–siRNA showed a heightened sensitivity to anthralin-mediated cell death due to enhanced loss of mitochondrial membrane potential [10]. This suggests that PHB might have a protective function against apoptosis. Increased PHB levels in response to subtle caloric restriction indicate that such an increase may be one of the early events leading to lifespan expansion in response to caloric restriction [11].

In normal renal tissue, PHB was reported to be positively expressed, but significantly down-regulated in renal biopsy specimens, and negatively correlated with the expression of alpha-smooth-muscle actin (α-SMA) and with the degree of tubulointerstitial lesions [12]. PHB is distinctly present in interstitial and tubular epithelial cells, and is significantly down-regulated in response to cellular injury. Furthermore, PHB expression is negatively correlated with the degree of tubulointerstitial lesions; the higher the degree of damage, the lower the expression of PHB. Additionally, the expression of PHB in kidney tissue of aristolochic acid-induced nephropathy rats was shown to be reduced [13]. Our previous studies [14], [15] demonstrated that a reduction in PHB expression was associated with a significantly increased manifestation of RIF and concomitant expression of TGF-β1, Col-IV, and FN. Moreover, enhanced oxidative stress seems to be associated with PHB expression. In endothelial cells, down-regulation of PHB resulted in increased mitochondrial reactive oxygen species (ROS) production and cellular senescence [16], whilst over-expression of PHB in intestinal epithelial cells ameliorated oxidative stress in inflammatory bowel disease [17]. Despite these intriguing results, PHB's exact role remains controversial. In different cell and tissue types, PHB is expressed to various degrees, *e.g.*, the PHB content is shown to be increased in a large number of cancers, but reduced in kidney disease. It is therefore difficult to state whether PHB is a protective or a risk factor in the pathogenesis of RIF.

To clarify the present turbid picture regarding PHB's role in RIF still requires a significant research effort. To the best of our knowledge, no study is available that reports on the relationship between PHB, oxidative stress, and the pathogenesis of RIF in the kidney. Therefore, this study was performed to explore what role the expression of PHB plays in the development of RIF in a well-characterized animal model, *i.e.*, unilaterally ureteral obstructed rats. It is expected that the insight gained will at least partially clarify PHB's role in RIF and might provide the basis for novel therapeutic interventions to countermand RIF in human patients

## Materials and Methods

### Animal model and gene interference *in vivo*

Healthy male Wistar rats (80∼100 g) were obtained from the Experimental Animal Center of Guangxi Medical University (Nanning, China). Rats were maintained at ∼22°C under a 12-h dark/light cycle, and were given food and water *ad libitum*. All experimental procedures involving lab animals were reviewed and approved by the animal ethics committee of Guangxi Medical University. The rats were randomly divided into five groups: (i) sham operation group (SHO); control group that underwent exactly the same surgical procedure as the experimental group, without the treatment under study, (ii) model group subjected to unilateral ureteral obstruction (UUO), (iii) UUO rats treated with lentivirus carrying PHB-siRNA (PHB^−^), (iv) UUO rats treated with lentivirus carrying *Phb* (PHB^+^), and (v) UUO rats treated with control viruses, *n* = 10, respectively. The lentivirus vectors, PHB^−^ and PHB^+^, and control viruses were purchased from Life Technologies-Invitrogen (Grand Island, NY, USA). Rats in the PHB^−^, PHB^+^, and negative control virus groups were injected intraperitoneally from the second day after surgery for three consecutive days. Subsequently, ten rats from those five groups were sacrificed 96 hours after the first injection and their renal tissue was collected for further experimental assessment.

### Microscopic assessment of renal morphology

Renal morphological assessment was essentially performed as described previously [14]. Briefly, the renal tissue was fixed (10% neutral formaldehyde), dehydrated via a graded ethanol series, embedded in paraffin, and microtome- prepared sections were subsequently stained with Masson's trichrome staining. Renal morphology and pathology was evaluated by optical microscopy. Blue granular or linear deposits were interpreted as positive areas for collagen staining and the severity of the renal lesions was expressed as the renal interstitial fibrosis (RIF) index. Semi-quantitative evaluation was performed by computer-assisted image analysis on a Leica DMR+Q550 system (Leica Microsystems GmbH, Wetzlar, Germany) at 400× magnification. The area of positive staining for fibrosis was measured in twenty fields (fields containing glomerular parts were ignored), which were randomly selected from coded sections for each rat [15]. RIF was scored as follows: 0 = absent (*n*_0_), 1 = less than 25% of the area (*n*_1_), 2 = 26 to 50% of the area (*n*_2_), and 3 = greater than 50% of the area (*n*_3_) [18]. The sections were evaluated blind by two investigators and the obtained scores were averaged. The RIF index was calculated according to:
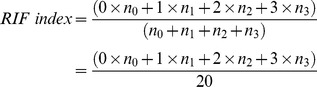



Additionally, Sirius Red staining for evaluating fibrosis was performed according to the method reported by Botto et al. [19]. Simultaneously, Col-I and III were stained and counter-coloration with hematoxylin of the cell nuclei (blue) allowed easy morphological assessment and resulted in a contrast enhancement.

### Immunohistochemical analysis of renal protein expression

Fixed renal tissue (4% buffered paraformaldehyde) was embedded in paraffin and 4 µm thick sections were subjected to immunohistochemical staining for PHB, TGF-β1, Col-IV, and FN. To recover antigenicity, the sections were deparaffinised by microwave treatment (10 mmol/L sodium citrate buffer, 10 min), washed with PBS, and treated with 3% H_2_O_2_ in methanol for 10 min. Subsequently, all sections were incubated with anti-prohibitin (1∶500), anti-TGF-β1 (1∶500), anti-Col-IV (1∶100), anti-fibronectin (1∶200) primary antibodies (all from Abcam, Cambridge, MA, USA), respectively, and visualized after incubation with secondary antibody and staining with diaminobenizidine. Positive areas were measured quantitatively using a computer-aided manipulator on a Leica DMR+Q550 system (fields containing glomerular parts were ignored). All evaluations were performed blind by two investigators.

### Real time reverse transcription polymerase chain reaction to detect PHB/, TGF-β1 mRNA expression

Renal tissue was homogenized and total RNA was extracted with TRIzol (Life Technologies-Invitrogen, Grand Island, NY, USA). Signals was measured with a Gel Doc XR+, UV/*vis*-Molecular Imager (Bio-Rad Laboratories, Hercules, CA, USA) and evaluation of the 18S and 28S RNA bands after agarose gel electrophoresis confirmed that there had been no RNA degradation [20]. Primers were designed according to standard primer design principles with Primer Premier 5.0 (Premier Biosoft, Palo Alto, CA, USA): PHB = F 5′–TGGCGTTAGCGGTTACAGGAG–3′ and R 5′-GAGGATGCGTAGTGTGATGTTGAC-3′; TGF-β1 = F 5′-TGAGCACTGAAGCGAAAGCC-3′ and R 5′-GATTCAAGTCAACTGTGGAGCAAC-3′; β-actin = F 5′-GCCCCTGAGGAGCACCCTGT-3′ and R 5′-ACGCTCGGTCAGGATCTTCA-3′.

One microgram total RNA from the renal tissue of each rat was reverse transcribed into cDNA with an ExScript RT reagent kit (Thermo Scientific-Fermentas, Waltham, MA, USA). PHB and TGF-β1 were amplified with SYBR Premix Ex Taq (Roche Inc., Basel, Switzerland). As an internal loading control, gene expression of the housekeeping β-actin (*ACTB*) was used as an internal loading control and to determine reverse transcription efficiency. The average threshold cycle (Ct; the cycles of template amplification to the threshold) was determined for each sample and the fold change in the data was analyzed according to [21]:
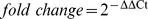
For example, the ΔΔCt for PHB mRNA expression in the UUO group was as follows:
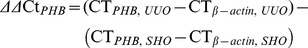
Subsequently, the fold change for PHB mRNA expression in the UUO group could be calculated from 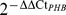.

### Western-blot Analysis

Proteins were isolated from homogenized renal tissue with radio immunoprecipitation assay (RIPA) lysis buffer (Sigma-Aldrich Corp., St. Louis, MO, USA) containing 0.25 nM of the protease inhibitor phenylmethanesulfonyl fluoride (PMSF; Sigma-Aldrich Corp., St. Louis, MO, USA). After protein concentration, quantization was performed with the modified Bradford assay (Bio-Rad Laboratories, Hercules, CA, USA) [22] and 40 mg total protein was subsequently used for Western blotting with primary antibodies against PHB, TGF-β1, Col-I, Col-III, Col-IV, and FN; β-actin was used as an internal loading control. Near-infra red fluorescence from manually selected bands of interest were imaged with an Odyssey Fc scanner (Li-Cor, Lincoln, NE, USA); Raw fluorescence intensities were background subtracted (intra-lane) using Li-Cor Odyssey 3.0 analytical software [23].

### Evaluation of cell apoptosis in renal tissue

Apoptosis was examined via TdT-mediated dUTP nick end-labelling (TUNEL; Roche Inc., Basel, Switzerland), as described previously [24], [25]. In brief, six slides from each kidney were evaluated microscopically for the percentage of apoptotic cells: on each section 20 watch fields (glomerular parts were excluded) were randomly chosen and evaluated. Brown staining of cell nuclei was considered positive for apoptotic cells and the apoptosis index was calculated according to [25]:
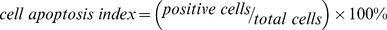
The scores obtained by two investigators were averaged.

### ROS, Lipid peroxidation, and antioxidant measurements

ROS measurements were essentially performed according to Hempel *et al.* [26], with some modifications. Excised renal tissue was washed, weighed, and subsequently homogenized in ice cold 0.9% normal saline solution. The homogenate was centrifuged at 4000×g for 15 min at 4°C and 500 µl supernatant was incubated for 3 h at 37°C with 10 µL of a 10 µM DCF-DA (2,7-dichlorodihydro-fluorescein diacetate) solution (Life Technologies/Molecular probes, Eugene, OR, USA). The fluorescence signal was measured at 485/525 nm on a S-3100 spectrofluorometer (Scinco Co. Ltd., Seoul, Korea), equipped with a 1024 channel photodiode array detector and expressed as arbitrary units.

Malonyldialdehyde (MDA), glutathione (GSH), and Superoxide Dismutase (SOD) were determined as extensively described by Wu *et al.* [27]. Kidney tissue was homogenized in 0.1 M phosphate buffer (pH 7.4) using a Ultra Turrax–T18–basic homogenizer (IKA Works, Inc. Wilmington, NC, USA), and the homogenate was centrifuged at a 10,000×g at 4°C for 15 min to remove cellular debris. The supernatant was used for the estimation of MDA and GSH levels, and SOD activity. Protein concentration of samples was determined with BCA (bicinchoninic acid) Protein assay kit (Sigma-Aldrich Corp., St. Louis, MO, USA) with BSA as a standard. Absorbance was measure spectrophotometrically at 562 nm.

### Statistical analysis

All data are shown as mean ± standard deviation (SD). To compare the groups in relation to parameters with normal distribution, one-way analysis of variation (ANOVA) with post-hoc Fisher's LSD (least significant difference) was used. Conversely, for those parameters without normal distribution, Kruskal-Wallis with post-hoc Mann-Whitney (only for the weight parameter) was used. Pearson's correlation coefficients were used to determine the relationships between the indicators for detection in the animal experiments. A value of P<0.05 was accepted as statistically significant. Statistical analysis was performed using the Statistical Package for Social Sciences–SPSS, version 13.0 (SPSS Inc., Chicago, IL, USA).

## Results

### Prohibitin expression and fibrosis

Various urinary tract obstructions are a major cause of progressive renal disease, especially congenital urinary tract obstruction in infants. Since unilateral ureteral obstruction (UUO) is a well-characterized model for experimental obstructive nephropathy [28], which causes amongst others oxidative stress and ultimately culminates in renal tubular apoptosis and interstitial fibrosis [29], [30], [31], [32], we opted to perform our experiments in lab animals accordingly. Lentivirus-based vectors were used to deliver either an interfering RNA to knock-down PHB protein expression or the *Phb* gene to attain exactly the opposite effect. Male Wistar rats (80∼100 g) were randomly divided into five groups: (i) sham operation group (SHO); control group that underwent exactly the same surgical procedure as the experimental group, without the treatment under study, (ii) model group subjected to unilateral ureteral obstruction (UUO), (iii) UUO rats treated with lentivirus carrying PHB-siRNA (PHB^−^), (iv) UUO rats treated with lentivirus carrying *Phb* (PHB^+^), and (v) UUO rats treated with control viruses.

**Figure 1:**
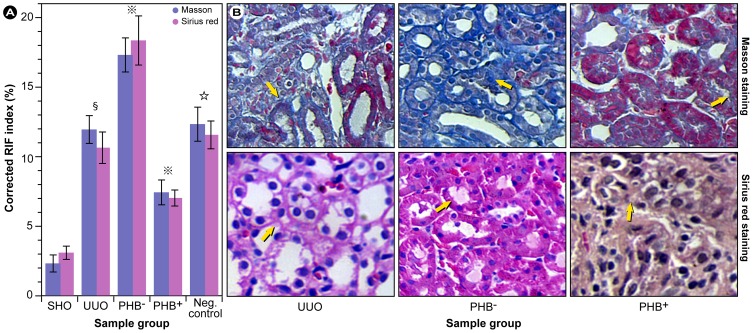
Evaluation of renal collagen deposition using Masson's trichrome stain. (Blue: collagen [arrow], Pink: cytoplasm; Brown-dark blue: nuclei) and Sirius red stain counter-colored with hematoxylin, (Pink: collagen [arrow], Blue: nuclei). (**A**) The extent of the renal lesions is represented by the RIF (renal interstitial fibrosis) index. **§**: *P*<0.01 compared with SHO group, **???**: *P*<0.01 compared with UUO group, **☆**: *P*>0.05 compared with UUO group. (**B**) Representative samples of Masson and Sirius red staining for SHO (sham operation), UUO (model group subjected to unilateral ureteral obstruction), PHB^−^ (UUO rats treated with lentivirus carrying siRNA PHB), and PHB^+^ (UUO rats treated with lentivirus carrying PHB) groups; Negative control: UUO rats treated with control viruses.

Microscopic observation of Masson-stained renal slices showed that more collagen was deposited in the renal interstitium of the UUO group when compared with the SHO control group. This is well reflected in the difference in the RIF index, as shown in Fig. 1A (*P*<0.01). The collagen deposition in the PHB^−^ group was notably increased, whilst in the PHB^+^ group such a deposition was reduced compared with the UUO group (Fig. 1). The observed differences between control viruses and UUO groups were statistically not significant (*P*>0.05; Fig. 1A), which excludes any interference or impact on the observation by the viral vector. Similar results were obtained when using Sirius red for collagen staining, albeit that the effect of PHB knock-down is more apparent in these micrographs.

### Expression of prohibitin and extracellular matrix components

Since the results from the Masson and Sirius red stainings suggested that increased PHB expression caused less deposition of ECM, further analysis of PHB levels, proteins involved in the ECM, i.e., collagen IV (Col-IV) and fibronectin (FN), and the profibrotic factor transforming growth factor beta 1 (TGF-β1) were performed. The latter was scrutinized because TGF-β1 is regarded to be a central mediator of interstitial fibrosis. Its levels both affect pro- and anti-fibrotic signalling, e.g., through the Smad or jagged/notch pathway.

**Figure 2:**
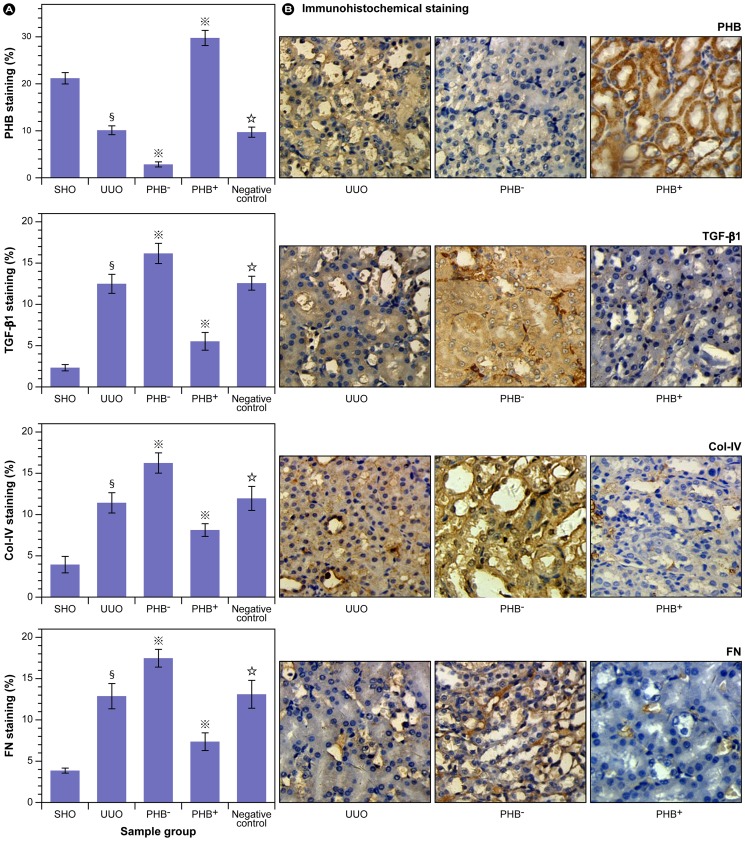
Immunohistochemical evaluation of protein expression involved in RIF. (**A**) Quantitative evaluation of the extent of the immunohistochemical staining for PHB, TGF-β1, Col-IV, and FN. **§**: *P*<0.01 compared with SHO group, **???**: *P*<0.01 compared with UUO group, **☆**: *P*>0.05 compared with UUO group. (**B**) Representative samples of immunohistochemical staining for PHB, TGF-β1, Col-IV, and FN in SHO (sham operation), UUO (model group subjected to unilateral ureteral obstruction), PHB^−^ (UUO rats treated with lentivirus carrying siRNA PHB), and PHB^+^ (UUO rats treated with lentivirus carrying PHB) groups; Negative control: UUO rats treated with control viruses.

Immunohistochemical staining of renal sections with appropriate antibodies (Fig. 2) clearly showed markedly less staining of PHB in the renal interstitium of UUO rats compared with SHO group rats (Fig. 2; top panel). Conversely, staining of TGF-βl was ∼6×, Col-IV, and FN ∼3× higher in UUO rat renal interstitium compared with SHO control (Fig. 2; *P*<0.01). As expected, PHB staining in the PHB^−^ group was reduced considerably compared with SHO control (by a factor of ∼7) and concomitantly TGF-βl, Col-IV, and FN content increased ∼4–5 times. Equally, PHB expression was ∼3 times higher in the PHB^+^ group compared with UUO rats. Furthermore, a large reduction in TGF-βl, Col-IV, and FN staining was observed. The differences in staining for all proteins between the UUO group and control viruses group were not significant (all *P*>0.05; Fig. 2).

**Figure 3:**
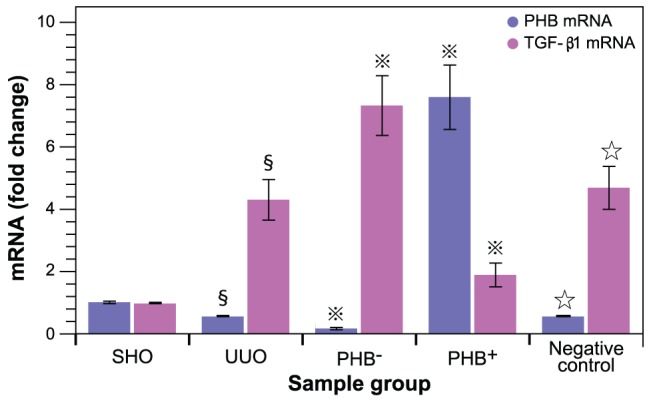
mRNA expression of PHB and TGF-β1 in RIF. **§**: *P*<0.01 compared with SHO (sham operation) group, **
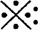
**: *P*<0.01 compared with UUO group (model group subjected to unilateral ureteral obstruction), **☆**: *P*>0.05 compared with UUO group. PHB^−^ (UUO rats treated with lentivirus carrying siRNA PHB); PHB^+^ (UUO rats treated with lentivirus carrying PHB) groups; Negative control: UUO rats treated with control viruses.

**Figure 4:**
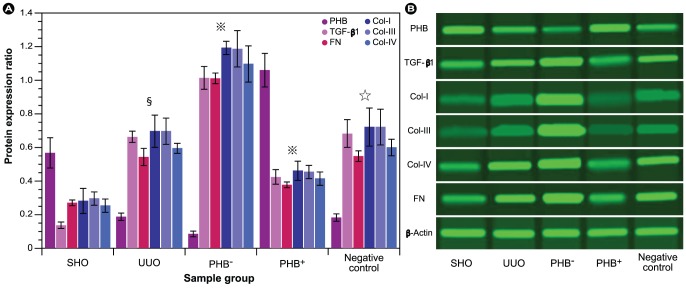
Evaluation of protein expression (Western-blot). (**A**) Protein ratios were calculated relative to β-actin (loading control). **§**: *P*<0.01 compared with SHO group, **
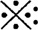
**: *P*<0.01 compared with UUO group, **☆**: *P*>0.05 compared with UUO group. (**B**) Representative Western blot for PHB, TGF-β1, Col-I, Col-III, Col-IV, and FN in SHO (sham operation), UUO (model group subjected to unilateral ureteral obstruction), PHB^−^ (UUO rats treated with lentivirus carrying siRNA PHB), and PHB^+^ (UUO rats treated with lentivirus carrying PHB) groups; Negative control: UUO rats treated with control viruses.

These results implicate that a significant knockdown or up-regulation of PHB expression can be achieved with the chosen experimental strategy. To further assess the expression of PHB and the potential changes in profibrotic factors in the five experimental groups, mRNA (Fig. 3) and protein expression (Fig. 4) of PHB and TGF-β1 were determined. Renal tissue from the UUO group showed consistently lower PHB mRNA expression, when compared to the SHO group (*P*<0.01). The desired knock-down of PHB-mRNA with the vector was nearly complete as shown in Fig. 3 (PHB^−^) and over-expression of PHB resulted in a two fold increase in PHB-mRNA (PHB^+^). Here, the gene interference and over-expression showed to be highly effective and were in conformity with the observed patterns in the immunohistochemistry. From the mRNA levels of TGF-β1, it may be concluded that reduction in PHB concomitantly promotes the formation of TGF-β1 and *vice versa*.

Lower PHB protein expression in UUO group was observed on Western blot analysis when compared to that in SHO group (*P*<0.01; Fig. 4). Similar results were obtained for PHB and proteins involved in fibrosis compared with the mRNA measurements (*vide supra*). Overall these results corroborate the results of the immunohistochemistry. In addition, we determined the expression of interstitial collagens, Col-I and Col-III, via Western blot analysis. The results in Fig. 4 show that both Col-I and Col-III were expressed similarly to Col-IV.

### The effect of prohibitin on oxidative stress and cell apoptosis

**Figure 5:**
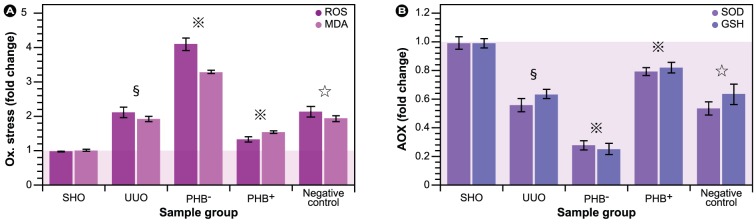
Assessment of oxidative stress and antioxidative capacity. Changes in general reactive oxygen species (ROS) levels and malondialdehyde (MDA) generation, a final degradation product of lipid peroxidation, are shown in (**A**) and superoxide dismutase (SOD) and reduced glutathione (GSH) in (**B**). **§**: *P*<0.01 compared with SHO group (sham operation), **
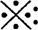
**: *P*<0.01 compared with UUO group (model group subjected to unilateral ureteral obstruction), **☆**: *P*>0.05 compared with UUO group. PHB^−^: UUO rats treated with lentivirus carrying siRNA PHB; PHB^+^; UUO rats treated with lentivirus carrying PHB; Negative control: UUO rats treated with control viruses; AOX: antioxidant.

Renal interstitial fibrosis is associated with accumulation of ECM components in the interstitium and oxidative stress via various initiators of RIF, e.g., obstructive ischemic hypoxia, or metabolic derangements in the fibrotic tissue. In order to assess the effect of PHB on oxidative stress, antioxidant status, and eventual apoptotic cell death, indicators of these in lentivirus-treated rats were measured. The results in figure 5A show that, as expected, oxidative stress was significantly larger in rats with urinary obstruction compared with SHO. Both ROS generation and malon dialdehyde (MDA) formation in the UUO group were much higher than control. Furthermore, the results in figure 5 clearly indicate that with decreasing PHB expression, ROS generation and MDA levels were significantly increased. Interestingly, PHB depletion had a significant effect on lipid peroxidation relative to the total ROS levels, which shows that biomembranes are prime targets during fibrotic processes.

Evaluation of endogenous antioxidants, i.e., superoxide dismutase (SOD) and glutathione (GSH) both confirmed a higher oxidative stress when PBH was knocked down (PHB^−^, Fig. 5B), since particularly GSH was significantly reduced. However, it also shows that SOD was compromised, which consequently lowers the cell's capability to dismutate the superoxide anion.

**Figure 6:**
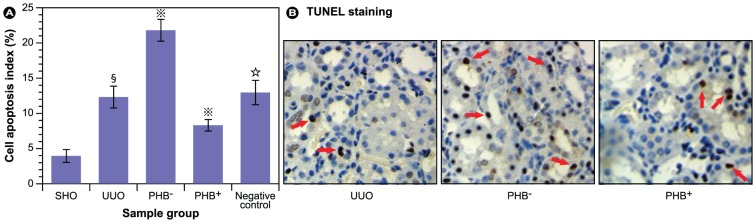
Evaluation of cell apoptosis (TUNEL assay). (**A**) Six slides from each kidney were evaluated for the percentage of apoptotic cells (brown nuclei) relative to all cells (brown and blue nuclei). **§**: *P*<0.01 compared with SHO group (sham operation), **
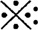
**: *P*<0.01 compared with UUO group, **☆**: *P*>0.05 compared with UUO group. (**B**) Representative micrographs of kidney tissue from UUO (model group subjected to unilateral ureteral obstruction), PHB^−^ (UUO rats treated with lentivirus carrying siRNA PHB), and PHB^+^ (UUO rats treated with lentivirus carrying PHB) groups; Negative control: UUO rats treated with control viruses.

Since such high levels of endogenous ROS and compromised antioxidants were observed and cells are lost even beyond tubular collapse via apoptosis in the acute phase of RIF, we evaluated if apoptosis could be attenuated by increasing PHB expression. The results in figure 6 show that the cell apoptosis index in the UUO group was much higher than the control group, which shows that urinary tract obstruction *per se* causes irreparable damage. In concordance with the previous experiments, the results in figure 6 show that over-expression of PHB significantly reduces apoptotic cell death in the cell population. Overall, the observed effects in the control viruses group were similar to the UUO group (*P*>0.05).

### Correlation analysis

Correlation analysis showed that PHB protein levels were negatively correlated with ROS, MDA, TGF-βl, Col-IV, FN, RIF and cell apoptosis index (*r* = −0.744, −0.832, −0.806, −0.783, −0.813, −0.903, 0.882; all *P*<0.01), but positively correlated with SOD and GSH (*r* = 0.889, 0.828; each *P*<0.01). Overall, all experiments and the correlation analysis suggest that PHB prevents oxidative stress, ECM accumulation and concomitant fibrosis in the rat model.

## Discussion

Renal hypoxia is considered a fundamental factor that contributes to the development of tubular atrophy and interstitial fibrosis, which in turn are both manifestations of renal disease progression and ultimately lead to renal failure [33], [34], [35]. Oxidative stress has long been implicated in a myriad of degenerative diseases and is, amongst others, associated with disruption of the redox state, damage to biomolecules, mitochondrial swelling, loss of mitochondrial integrity, and cell apoptosis or necrosis. Such damaging processes can be counteracted by compounds that inhibit, sequester, or dismutate the damaging reactive or catalyzing species. Increasingly evidence emerges that abrogation of oxidative stress might be a protective strategy to prevent renal degenerative diseases [36], [37], [38]. However, virtually no reports are available that link oxidative stress with the pathogenesis of RIF and therefore the current study was performed to explore if causal associations exist. It is well established that TGF-β1 plays a significant pathogenic role in renal fibrosis [1], [39], [40] and, most significantly, is able to induce the deposition and accumulation of ECM components (Col-I, Col-III, Col-IV, and FN) [3]. The aforementioned factors are important indicators to evaluate the severity of the occurring RIF lesions. PHB can readily be detected in renal tissue and has been associated with the development of RIF. However, whether PHB plays a protective or rather degenerative role in RIF remains obscure.

In this investigation, we tried to answer this question by studying the effect of PHB in a RIF rat model (UUO). Our experimental results show that ROS and MDA as indicators of oxidative stress were increased and that SOD and GSH as indicators for the antioxidative status were concomitantly reduced in the UUO group compared with the control group. Furthermore, PHB mRNA and protein expression were significantly reduced in the UUO group compared with control and concomitantly TGF-βl, Col-I, Col-III, Col-IV, and FN were up-regulated. We therefore hypothesized that PHB might act as an antioxidant or at least induce protective mechanisms that reduce oxidative damage in RIF.

In order to further elucidate this notion, we performed additional studies with UUO rats using gene interfering to reduce the intracellular PHB content. A reduction of PHB resulted in increased ROS and MDA formation on the one hand, and a reduction in SOD and GSH on the other. Conversely, overexpression of PHB via lentivirus transfection of *Phb* distinctly reduced ROS and MDA and increased SOD and GSH. Since GSH is such a potent antioxidant, the observation that at least its normal levels are maintained when PHB is over-expressed is biologically highly relevant. PHB mRNA and protein expression in the PHB^−^ group were notably down-regulated compared with those in the UUO group. In addition, we found that mRNA and protein expression of TGF-βl, Col-I, Col-III, Col-IV, and FN in the PHB^−^ group were all up-regulated compared with the UUO group. In the PHB^+^ group, mRNA and protein expression were down-regulated. Overall, we found that PHB protein expression was negatively correlated with the RIF index, ROS, MDA, TGF-βl, Col-IV, FN, and cell apoptosis index.

The observations in the current study are largely in agreement with results reported by others. For instance, Suzuki Y. *et al.* [41] recently reported that the expression of TGF-β1 was increased in renal tissue of ureteral obstruction rats, which our study corroborates. Furthermore, several studies report the observation that Col-IV and FN accumulate in the tubulointerstitial of UUO rats or mice [42], [43], [44], [45]. Even though the exact mechanism by which PHB counteracts oxidative stress is unknown, we speculate whether this positive action might be induced by: (i) an active maintenance of biomembrane integrity. In yeast, PHB is known to act as a protein and lipid scaffold in the inner mitochondrial membrane to maintain its integrity; (ii) stabilization of respiratory chain proteins via PHB's chaperone function might reduce ROS formation by maintaining the function of complex I; (iii) PHB might exert a direct stabilization of antioxidant proteins; (iv) as stated previously, GSH levels were retained when PHB was over-expressed. Since nuclear PHB is known to modulate transcription initiation [8], [46], it is conceivable that PHB binds to antioxidant response elements [47] in the promoter of multiple antioxidant enzyme genes and up-regulates antioxidant gene expression.

In conclusion, we report that an increased PHB expression down-regulates ROS and MDA levels, and increases the expression of SOD and GSH *in vivo*. Concomitantly, TGF-βl, Col-I, Col-III, Col-IV, and FN expression is down-regulated and consequently ECM deposition is attenuated. Furthermore, this study is also the first to show that an increase in PHB reduces cellular apoptosis in renal tubulointerstitial lesions in UUO rats. Prohibitin therefore acts as a positive regulator of mechanisms that counteract oxidative stress and ECM accumulation. These results collectively suggest that maintaining at least near normal physiological levels of PHB in renal tissue ensures that the capacity to counteract the effects of oxidative stress is retained. If PHB expression is compromised, damaging and fibrotic processes are given free hand. Future investigations will have to show whether the introduction of PHB in already fibrotic tissue will reverse the damaging effect. Alternatively, a therapeutic strategy with PHB in the early stages of the fibrotic disease might retard its progression and alleviate the patient's symptoms. Our experimental results in UUO rats show that with such a therapeutic strategy, at least (partial) recovery might be conceivable. Further evidence for at least partial reversal of RIF was recently shown by Chevalier's group, who reported that in neonatal mice, relief of partial UUO arrested glomerulotubular disconnection, resolved tubule atrophy, and interstitial fibrosis [48]. Nonetheless, the translational potential of PHB expression modulation requires a significantly better understanding of the role of PHB in the mechanisms that lead to RIF before effective clinical applications are possible.
